# Activity of Pterostilbene Metabolites against Liver Steatosis in Cultured Hepatocytes

**DOI:** 10.3390/molecules25225444

**Published:** 2020-11-20

**Authors:** Jenifer Trepiana, Stéphanie Krisa, María Puy Portillo

**Affiliations:** 1 Nutrition and Obesity Group, Department of Nutrition and Food Science, Faculty of Pharmacy, University of Basque Country (UPV/EHU) and Lucio Lascaray Research Center, 01006 Vitoria-Gasteiz, Spain; jenifer.trepiana@ehu.eus; 2 BIOARABA Institute of Health, 01009 Vitoria-Gasteiz, Spain; 3 CIBEROBN Physiopathology of Obesity and Nutrition, Institute of Health Carlos III (ISCIII), 28029 Vitoria-Gasteiz, Spain; 4 Université de Bordeaux, UR Œnologie, MIB, EA 4577, USC 1366 INRA, 33882 Villenave d’Ornon, France; stephanie.krisa@u-bordeaux.fr

**Keywords:** pterostilbene metabolites, pterostilbene-4′-*O*-glucuronide, pterostilbene-4′-*O*-sulfate, liver steatosis, AML-12 hepatocytes

## Abstract

Pterostilbene is a dimethyl ether derivative of resveratrol, less metabolized than its analogue, due to the substitution of two hydroxyl groups with methoxyl groups. Nevertheless, the amounts of pterostilbene phase II metabolites found in plasma and tissues are higher than those of the parent compound. The first aim of this study was to assess whether pterostilbene-4′-*O*-glucuronide (PT-G) and pterostilbene-4′-*O*-sulfate (PT-S) were able to prevent triglyceride accumulation in AML12 (alpha mouse liver 12) hepatocytes. This being the case, we aimed to analyze the mechanisms involved in their effects. For this purpose, an in vitro model mimicking the hepatocyte situation in fatty liver was developed by incubating mouse AML12 hepatocytes with palmitic acid (PA). For cell treatments, hepatocytes were incubated with 1, 10 or 25 µM of pterostilbene, pterostilbene-4′-*O*-glucuronide or pterostilbene-4′-*O*-sulfate for 18 h. Triglycerides and cell viability were assessed by a commercial kit and crystal violet assay, respectively. Protein expression of enzymes and transporters involved in triglyceride metabolism was analyzed by immunoblot. The results showed for the first time the anti-steatotic effect of pterostilbene metabolites and thus, that they contribute to the preventive effect induced by pterostilbene on steatosis in in vivo models. This anti-steatotic effect is mainly due to the inhibition of de novo lipogenesis.

## 1. Introduction

Stilbenes have gained remarkable significance in nutrition research due to their beneficial effects on health [1]. Among them, resveratrol (*trans*-3,5,4-trihydroxystilbene) is the most extensively studied example. It has shown many positive biological effects against cancer, oxidative stress and inflammation [2,3]. Its low bioavailability, due to the extensive phase II metabolism that it occurs in intestine and liver [4,5,6], has prompted two interesting lines of research: analyzing the potential biological activities of its metabolites, and investigating resveratrol-derived stilbenes with higher bioavailability.

With regard to the first line of research, although to date little is known concerning this issue, several studies have shown that resveratrol metabolites show positive effects in cancer [7,8,9], neurodegenerative diseases [10] and type 2 diabetes mellitus [11], and also that they can act as anti-inflammatory agents [12]. In our group, we have reported that several resveratrol metabolites show delipidating effects in adipocytes and that they modify adipokine production [13,14,15]. More recently, we have observed that several metabolites also prevent hepatic steatosis (data submitted).

As far as the second line of research is concerned, pterostilbene (*trans*-3,5-dimethoxy-4′-hydroxystilbene) is a dimethyl ether derivative of resveratrol, less metabolized than its parent compound, due to the substitution of two hydroxyl groups with methoxyl groups [6]. This feature increases its transport into cells, as well as its metabolic stability. Consequently, its bioavailability is higher than that of resveratrol, as demonstrated in several studies [16,17], and thus it has been shown that pterostilbene plasma levels are higher than those of resveratrol when administered at equimolar doses [16]. Nevertheless, in spite of this higher bioavailability, plasma concentrations of pterostilbene phase II metabolites are much higher than is the concentration of the parent compound [16]. In line with these results, in a previous study from our group, we observed that after the administration of 30 mg/kg body weight/d of pterostilbene to rats, the proportion of pterostilbene, pterostilbene-4′-*O*-glucuronide and pterostilbene-4′-*O*-sulfate in liver was 1:2:9 (data submitted). Consequently, as in the case of resveratrol, it is important to analyze the potential activity of pterostilbene phase II metabolites because they could contribute importantly to the effects of the parent compound. Regarding this issue, as far as is known, only one recent study has been published about the neuroprotective and anti-inflammatory effects of pterostilbene phase II metabolites in human neuroblastoma cells and macrophages [18].

Taking all of the above into account, the first aim of the present study was to assess whether the two main pterostilbene phase II metabolites, pterostilbene-4′-*O*-glucuronide (PT-G) and pterostilbene-4′-*O*-sulfate (PT-S), were able to prevent triglyceride accumulation in AML12 (alpha mouse liver 12) hepatocytes. This being the case, the second aim was to analyze the mechanisms involved in their effects.

## 2. Results

### 2.1. Effects on Triglyceride Accumulation

**Figure 1:**
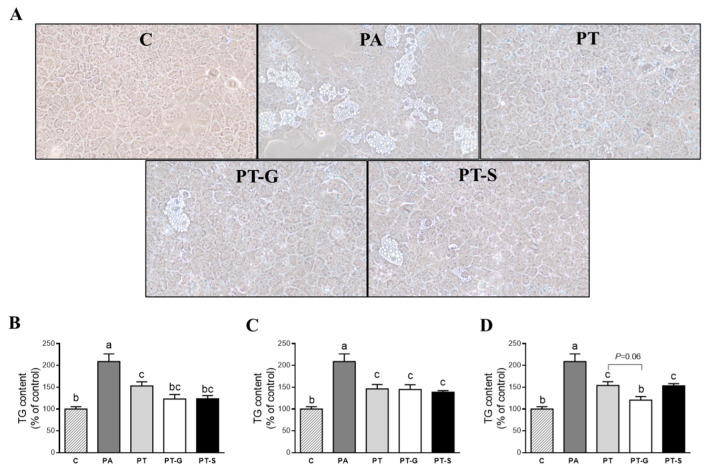
Optical microscopy images showing structural features at 1 µM (**A**), and triglyceride content in AML12 (alpha mouse liver 12) hepatocytes exposed to 0.3 M palmitic acid (PA) with or without pterostilbene (PT) or its metabolites (PT-G and PT-S) at 1 µM (**B**), 10 µM (**C**) or 25 µM (**D**) for 18 h. Data are means ± SEM. Values not sharing a common letter (a, b and c) are significantly different (*p* < 0.05). At 1 µM: C (control group) vs. PA, *p =* 0.000; C vs. PT, *p* = 0.000; PA vs. PT, *p* = 0.04; PA vs. PT-G, *p* = 0.002; PA vs. PT-S, *p* = 0.001. At 10 µM: C vs. PA, *p* = 0.000; C vs. PT, *p* = 0.004; C vs. PT-G, *p* = 0.009; C vs. PT-S, *p* = 0.000; PA vs. PT, *p* = 0.03; PA vs. PT-G, *p* = 0.028; PA vs. PT-S, *p* = 0.005. At 25 µM: C vs. PA, *p* = 0.000; C vs. PT, *p* = 0.000; C vs. PT-S, *p* = 0.000; PA vs. PT, *p* = 0.04; PA vs. PT-G, *p* = 0.001; PA vs. PT-S, *p* = 0.038; PT-G vs. PT-S, *p* = 0.017.

To analyze the effects of pterostilbene and its metabolites, optical analysis was carried out. In addition, in order to have more accurate results, the amounts of triglycerides accumulated in the hepatocytes were spectrophotometrically quantified. At 1 µM, cells treated with pterostilbene or its metabolites showed reduced triglyceride accumulation (PT −27%, PT-G −41%, PT-S −41% vs. PA group). The triglyceride amount observed in the control cells was reached in cells treated with both metabolites, but not with pterostilbene (Figure 1B). At 10 µM, cells treated with pterostilbene or its metabolites showed similar reductions in triglycerides (Figure 1C). When hepatocytes were treated with the compounds at 25 µM, a trend to reduced amounts of triglycerides was observed in the PT-G group compared to that treated with the parent compound (PT-G group vs. PT group; *p* = 0.06; Figure 1D). At this concentration, no significant differences were observed between PT and PT-S.

Optical microscopy analysis showed that the treatment with PA alone displayed macrovacuolar steatosis in AML12 hepatocytes, when compared with the non-steatotic cells, where no lipid droplets were observed (Figure 1A). Lipid accumulation in cells co-incubated with PA and PT or its metabolites at 1 µM appeared as scattered smaller fat vacuoles in the cytoplasm of the AML12 (Figure 1A).

### 2.2. Cell Viability

**Figure 2:**
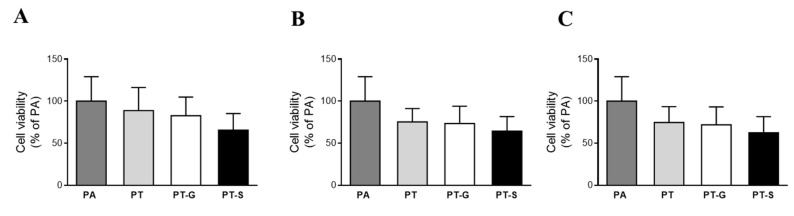
Cell viability in AML12 hepatocytes exposed to 0.3 M palmitic acid (PA) with or without pterostilbene (PT) or its metabolites (PT-G and PT-S) at 1 µM (**A**), 10 µM (**B**) or 25 µM (**C**) for 18 h. Data are means ± SEM.

The incubation of hepatocytes with pterostilbene or its metabolites for 18 h did not statistically decrease cell viability in the whole range of the concentrations studied, compared with the PA group (Figure 2A–C). It is worth mentioning that the incubation of AML12 hepatocytes with PA alone reduced the cell viability (−39%) when compared with the control group (non-steatotic cells; data not shown).

### 2.3. Effects of Pterostilbene and Pterostilbene Metabolites on Proteins Involved in Triglyceride Metabolism

In order to elucidate the mechanisms involved in the delipidating effect of the compounds analyzed, protein expression of key proteins involved in lipid metabolism was assessed by immunoblot. Because the lowest concentration of PT and its metabolites showing delipidating effect was 1 µM, this was the dose used for the immunoblot analysis.

**Figure 3:**
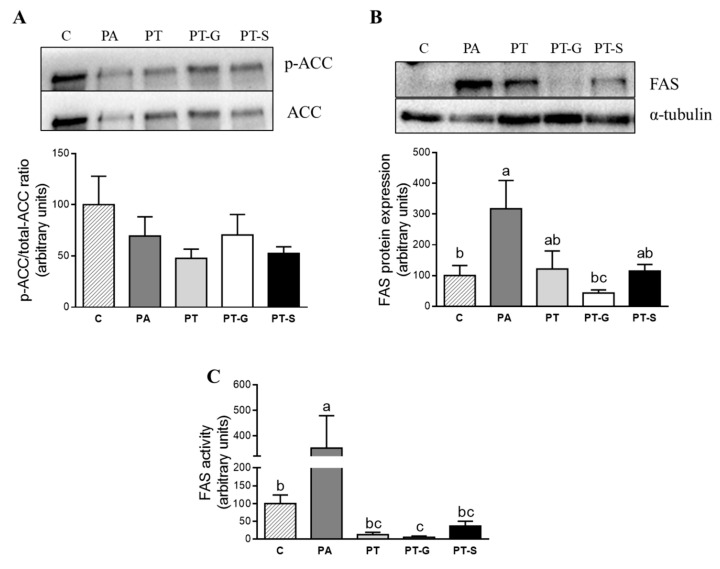
p-ACC/total-ACC ratio (index of ACC activity) (**A**), protein expression of FAS (**B**), and FAS activity (**C**) in AML12 hepatocytes incubated with 0.3 M palmitic acid (PA), with or without pterostilbene (PT) or its metabolites (PT-G and PT-S), for 18 h. The Western blot bands shown are representative of 6 samples/group. FAS protein expression was normalized by α-tubulin. Data are means ± SEM. Values not sharing a common letter (a, b and c) are significantly different (*p* < 0.05). In FAS protein expression: C (control group) vs. PA, *p* = 0.045; PA vs. PT-G, *p* = 0.022. In FAS activity: C vs. PA, *p* = 0.04; C vs. PT-G, *p* = 0.029; PA vs. PT; *p* = 0.003; PA vs. PT-G, *p* = 0.001; PA vs. PT-S, *p* = 0.039. ACC: Acetyl-CoA carboxylase; FAS; fatty acid synthase.

Taking into account that Acetyl-CoA Carboxylase (ACC) is inactivated by phosphorylation, the ratio phosphorylated-ACC/total-ACC is considered as an index of ACC activity (lower ratio meaning the activation of the enzyme). The incubation of hepatocytes with PA alone or in combination with pterostilbene or its metabolites did not affect the activity of ACC (Figure 3A). As far as FAS is concerned, PA induced a great increase in protein expression of this enzyme. This effect was partially blocked in cells co-incubated with pterostilbene or PT-S, and totally blocked in cells co-incubated with PT-G (Figure 3B). In light of these results, FAS activity was also measured. In this case, the effects followed a similar pattern, but they were more marked because the three sets of cells treated with PA and pterostilbene or its metabolites showed significantly lower values than cells only treated with PA (Figure 3C).

**Figure 4:**
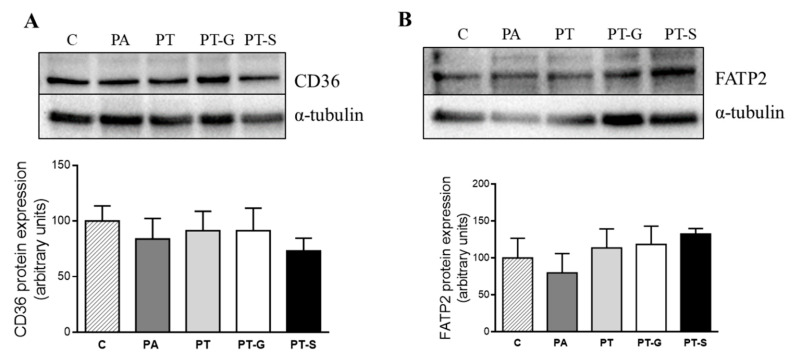
Protein expression of CD36 (**A**) and FATP2 (**B**) proteins in AML12 cells exposed to 0.3 M palmitic acid (PA), with or without pterostilbene (PT) or its metabolites (PT-G and PT-S) for 18 h. The Western blot bands shown are representative of 6 samples/group. CD36 and FATP2 protein expressions were normalized by α-tubulin. Data are means ± SEM. C: control group; CD36: fatty acid-transporter; FATP2: very long-chain acyl-CoA synthetase 1.

With regard to fatty acid uptake across the plasma membrane, CD36 and FATP2 proteins were analyzed. After 18 h of treatment, no significant changes in these fatty acid transporters were induced by PA. In hepatocytes co-treated with pterostilbene or its metabolites, protein expression of CD36 and FATP2 remained unchanged (Figure 4A,B).

**Figure 5:**
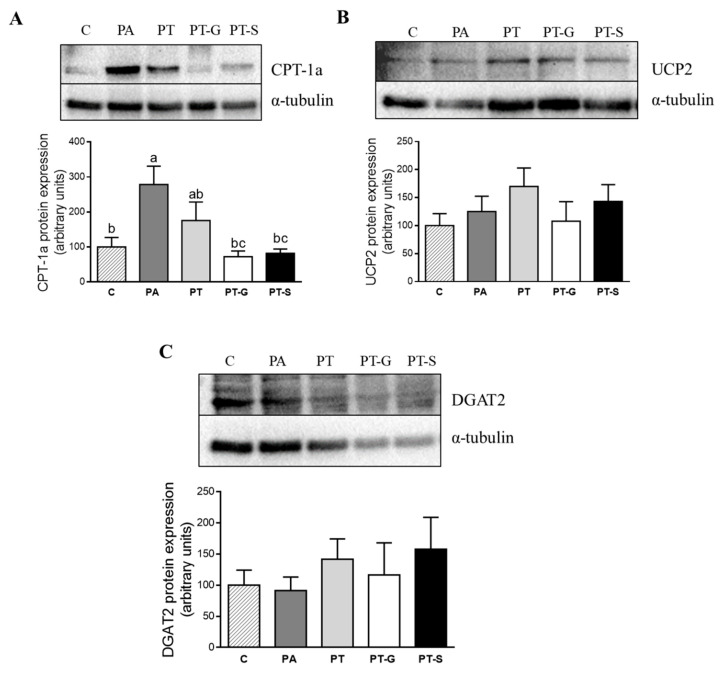
Protein expression of CPT-1a (**A**), UCP2 (**B**), and DGAT2 (**C**) in AML12 cells exposed to 0.3 M palmitic acid (PA), with or without pterostilbene (PT) or its metabolites (PT-G and PT-S) at 1 µM for 18 h. The Western blot bands shown are representative of 6 samples/group. CPT-1a, UCP2, and DGAT2 protein expressions were normalized by α-tubulin. Data are means ± SEM. Values not sharing a common letter (a, b and c) are significantly different (*p* < 0.05). In CPT-1a protein expression: C (control group) vs. PA, *p* = 0.012; PA vs. PT-G, *p* = 0.007; PA vs. PT-S, *p* = 0.011. CPT-1a: carnitine palmitoyl-transferase 1a; UCP2: mitochondrial uncoupling protein 2; DGAT2: diacylglycerol *O*-acyltransferase 2.

As far as fatty acid oxidation is concerned, PA significantly increased CPT-1a protein expression. In cells treated with pterostilbene, this effect was partially prevented, and totally prevented in cells treated with the metabolites. On the other hand, neither PA nor PT or its metabolites modified UCP2 protein expression (Figure 5B). Finally, to evaluate the influence of phenolic compounds on triglyceride assembly, protein expression of DGAT2 was assessed, but no significant differences were observed (Figure 5C).

## 3. Discussion

Pterostilbene presents an important advantage with regard to its analogue resveratrol, due to the presence of two methoxy groups instead of two hydroxyl groups in its chemical structure. It has been reported that UDP-glucuronosyltransferases UGT1A1 and UGT1A3 preferentially attack the 3-hydroxyl group of resveratrol [17]. On the other hand, the UDP-glucuronosyltransferase UGT1A9, which has the highest glucuronidation activity against the 4′-hydroxyl group of resveratrol, has minimal activity against the 4′-hydroxyl group of pterostilbene [19]. With regard to sulfotransferases, the 3-hydroxyl position is preferred by these enzymes to form the 3-*O*-sulfate, and sulfation at the 4′-hydroxyl position of resveratrol is significantly less efficient. Moreover, only SULT1E1 has demonstrated some specificity for the 4′-hydroxyl group [20]. These data suggest that the metabolic stability of pterostilbene is much higher than that of its analogue resveratrol, since the dimethoxy structure of pterostilbene restricts the glucuronidation/sulfation process only at the 4′ position, because the 3′ position is methylated. Nevertheless, as indicated in the Introduction Section, plasma concentrations and tissue amounts of pterostilbene phase II metabolites are much higher than those of the parent compound.

In this scenario, the anti-steatotic effect of pterostilbene metabolites was analyzed in the present study, in order to determine whether they can contribute to the effect of the parent compound on the prevention of liver steatosis observed in vivo [21]. In this regard, it was reported that the sulfate and glucuronide metabolites are the main ones in mice [22]. In rats, Azzolini et al. observed that pterostilbene-sulfate was present in all tissues 2 h after pterostilbene administration [23]. Indeed, in their study, the authors reported that the highest levels were detected in the liver, where pterostilbene-sulfate was approximately 5-fold higher than its parent compound. In agreement with this study, this trend was observed in an in vivo study carried out by our research group, using a model of liver steatosis induced by high-fat high-fructose feeding, where the most abundant metabolite present in the liver was pterostilbene-sulfate (data submitted). According to these results, pterostilbene-4′-*O*-sulfate (PT-S) and pterostilbene-4′-*O*-glucuronide (PT-G) were selected to be analyzed in the present study.

The doses of pterostilbene and its metabolites chosen in the present study were 10 and 25 μM because they are commonly used in in vitro studies, and also 1 μM because the concentrations found in plasma in animals supplemented with pterostilbene are similar to this value. In fact, Azzolini et al. reported that maximum blood concentrations of this phenolic compound 2 h after oral consumption were around 1.5 µM [23]. Moreover, it was described that PT-S reached the maximum concentration of 11.7 µM after administering pterostilbene at a dose of 22.5 mg/kg [23], whereas PT-G was present at about 1–2.3 µM [16,24].

Interestingly, when triglyceride accumulation was analyzed, the best results were obtained at the lowest dose (1 µM). At this dose, triglyceride accumulation induced by PA was partially prevented when hepatocytes were co-incubated with PA and pterostilbene, and totally prevented when they were co-incubated with PA and PT-G or PT-S. The effects observed in cells treated with both metabolites were greater than those found in cells treated with pterostilbene (−41% vs. −27%), although the differences were not statistically significant. These results show that phase II metabolites contribute to the preventive effect on liver steatosis observed in animals treated with this phenolic compound. Taking into account that, as reported in the literature, the amount of PT-S in liver is clearly higher than that of PT-G, it can be proposed that, although in vitro both metabolites show the same effectiveness, PT-S contributes in a greater extent to the effect of pterostilbene.

The fact that in cells treated with pterostilbene the reduction in triglyceride content was lower than in cells treated with the metabolites suggests that the parent compound is less effective than its metabolites. Nevertheless, taking into account that after 18 h of incubation the amount of pterostilbene inside the cells could be expected to be less than that of its metabolites, a higher effectiveness of pterostilbene cannot be ruled out. In order to solve this issue, it would be necessary to inhibit the sulfotransferase SULT1E1, which is responsible for the synthesis of the main pterostilbene phase II metabolite. In order to carry out this experiment, we revised the literature but did not find an inhibitor of this enzyme for rodents. Consequently, we can state that this issue is a limitation of our study.

For this reason, one limitation of the present study is that we cannot state whether pterostilbene metabolites are more effective than the parent compound.

In order to understand the mechanisms underlying the effects of pterostilbene metabolites, we analyzed several metabolic pathways involved in triglyceride accumulation. The treatment of AML12 hepatocytes with the saturated fatty acid PA induced a strong increase in protein expression and activity of FAS. It is worth noting that CPT-1a protein expression was upregulated in steatotic AML-12 cells. Consequently, we proposed that in hepatocytes treated with PA for 18 h, the activation of the β-oxidation is a compensatory mechanism devoted to increasing the transport of fatty acids into the mitochondrion for their subsequent oxidation, with the aim of reducing the steatosis development. With regard to these results, this mechanism has also been observed previously by our research group in an in vivo study carried out in an experimental model of liver steatosis induced by high-fat high-fructose feeding (data submitted). Pterostilbene metabolites totally prevented the increase induced by the diet in FAS activity. Thus, CPT-1a was not increased in AML12 cells treated with these compounds. To explain this result, we propose that the compensatory mechanism was no longer necessary.

On the other hand, in an attempt to obtain information concerning the transporting of fatty acids into the hepatocytes, protein expression of the transporters CD36 and FATP2 was analyzed [25], but no significant changes were observed. Overall, these results suggest that pterostilbene and its metabolites partially prevented steatosis due, at least in part, to a reduction in fatty acid availability for triglyceride accumulation, coming from their effects on de novo lipogenesis. Finally, concerning the triglyceride assembly, no changes were observed in DGAT2 protein expression by any of the treatments used in the present study.

This is the first time that the anti-steatotic effect of pterostilbene phase II metabolites has been reported, showing that they contribute to the preventive effect induced by pterostilbene on liver steatosis in animal models. This anti-steatotic effect is mainly due to the inhibition of de novo lipogenesis.

## 4. Materials and Methods

### 4.1. Reagents

Dulbecco modified Eagles minimal essential medium (DMEM)/HAM’s F12 (F-12 Nutrient medium) Glutamax, insulin, transferrin and selenium (ITS) were obtained from Thermofisher (Waltham, MA, USA). Fetal bovine serum (FBS) was purchased from Corning (New York, NY, USA). Streptomycin-penicillin solution and trypsin/EDTA were obtained from Lonza (Basilea, Suiza). Acetyl-coenzyme A, dexhametasona, malonyl coenzyme A, NADPH, palmitic acid (PA) and pterostilbene (≥97%), were purchased from Sigma-Aldrich (St Louis, MO, USA). Pterostilbene metabolites were kindly provided by Dr. Agnes Rimando from the University of Mississippi.

### 4.2. Cell Culture and Maintenance

Mouse hepatocyte AML12 (alpha mouse liver 12; ATCC^®^ CRL-2254™) was obtained from ATCC (Manassas, VA, USA). These cells were maintained in 75 cm^2^ flasks in DMEM/HAM’s F12 Glutamax supplemented with 10% heat inactivated fetal bovine serum plus 5 µg/mL insulin, 5 µg/mL transferrin, 5 ng/mL selenium, 40 ng/mL dexametasona and 1% penicillin/streptomycin (10,000 U/mL). AML12 were grown at 37 °C in a humidified atmosphere with 5% CO_2_. When the cell monolayer reached 75% of confluence, cells were detached with a solution of trypsin-EDTA, and then harvested to perform subsequent experiments.

### 4.3. Experimental Design

An in vitro model mimicking the hepatocyte situation in fatty liver was created by using mouse AML12 hepatocytes, which were grown in 6-well plates and incubated with 0.3 mM of PA for 18 h to induce triglyceride accumulation. To achieve the appropriate conditions for this in vitro model, toxicity and triglyceride levels time-course experiments were carried out (data not shown). In the groups treated with the phenolic compounds, hepatocytes were co-incubated simultaneously with PA and pterostilbene (PT), PT-G or PT-S at 1, 10 or 25 µM (diluted in 95% ethanol) along the 18 h of the experimental study. In the case of the control group, the same volume of the vehicle was used. After 18 h, cells were used for the subsequent experiments. Each experiment was performed at least three times.

### 4.4. Determination of Triacylglycerol Levels

After treatment, the medium was removed, and cell extracts were used for triglyceride determination. AML12 cells were washed extensively with phosphate-buffered saline (PBS), and the suspension was sonicated in 10 mM Tris-HCl pH 7.4, 150 mM NaCl and 1 mM EDTA on ice with five 5-s bursts in a Branson Sonifier SFX550 (San Luis, Misuri, MO, USA) fitted with a microtip. Furthermore, triglyceride content was measured with a commercial kit (Spinreact, Girona, Spain). Protein measurements were performed using the Bradford method [26]. Triglyceride content values were obtained as mg triglycerides/mg protein and expressed as the percentage of the control cells.

### 4.5. Cell Viability Assay

The live cell number was evaluated with the crystal violet assay based on cell staining with crystal violet [27]. Briefly, AML12 cells were seeded to 96-well tissue culture plates at 5 × 10^3^ cells per well, and three days after plating, the cells were treated with the pertinent compounds for 18 h. After treatments, cells were washed with PBS, fixed in 3.7% formaldehyde and stained with 0.25% crystal violet for 20 min in the dark. Finally, the resultant crystals were solubilized with 33% acetic acid and the absorbance was registered at 590 nm in an iMark microplate reader (Bio-Rad, Hercules, CA, USA). Cell viability was expressed as the percentage of the control cells.

### 4.6. Optical Microscopy Analysis of Steatotic AML12 Hepatocytes

Lipid droplet accumulation was analyzed by optical microscopy. AML12 cells seeded in 6-well culture plates and incubated with their respective treatments for 18 h were photographed under an Olympus CH optical microscope (Olympus, Tokyo, Japan) and examined with a 40× objective. The cell features were analyzed by ImageJ software (NIH, Bethesda, Maryland, MD, USA).

### 4.7. Protein Immunodetection

Phospho-acetyl-CoA carboxylase (p-ACC), total acetyl-CoA carboxylase (total-ACC), fatty acid synthase (FAS), carnitine palmitoyltransferase la (CPT-1a), mitochondrial uncoupling protein 2 (UCP2), diacylglycerol O-acyltransferase 2 (DGAT2), CD36 molecule (CD36), solute carrier family 27 member 2 (FATP2) and α-tubulin were detected by Western blot. Cellular protein extracts were denaturalized at 95 °C for 5 min in Laemmli buffer [28] and separated by sodium dodecyl sulfate polyacrylamide gel electrophoresis (SDS-PAGE) electrophoresis in 4–15% polyacrylamide gels. Gels were transferred onto polyvinylidene difluoride (PVDF) membranes by electroblotting with constant amperage (1 mA/cm^2^). After blocking for 1 h at room temperature, membranes were incubated overnight at 4 °C with the corresponding primary antibody (anti-p-ACC 1:1000, anti-total-ACC 1:1000, anti-FAS 1:1000, anti-CPT-1a 1:1000, anti-UCP2 1:1000, anti-DGAT2 1:1000, anti-CD36 1:1000, anti-FATP2 1:1000 and anti-α-tubulin 1:2000). After washing, membranes were probed with the secondary antibody conjugated to horseradish peroxidase. The immunoreactive proteins were detected with the Forte Western Horseradish Peroxidase (HRP) substrate (Millipore; Burlington, MA, USA) and the blots were imaged by scanning with the ChemiDoc™ MP Imaging System (Bio-Rad, Hercules, CA, USA). α-Tubulin was used as the loading control.

### 4.8. Fatty Acid Synthase (FAS) Activity

The samples for assaying the lipogenic fatty acid synthase activity were centrifuged at 5000× *g* for 5 min at 4 °C. The supernatant fraction was then used for quantification of enzyme activity fatty acid synthase from the rate of malonyl-CoA-dependent NADPH oxidation [29]. NADPH was measured by reading absorbance at 340 nm in an iEMS microplate reader (Lab systems; Bradenton, FL, USA). The enzyme assay was conducted at 37 °C. Soluble protein in the supernatant fraction was determined using bovine serum albumin as standard [26]. Fatty acid synthase (FAS) activity was expressed as nmol NADPH consumed/min per mg protein.

### 4.9. Statistical Analysis

Data were expressed as mean ± standard error of the mean (SEM) from at least three independent experiments. Normality of the data was tested using the Shapiro–Wilk test. Kruskal–Wallis, or one-way analysis of variance (ANOVA) followed by a Tukey´s or a Games-Howell´s post-hoc analysis were selected as appropriate according to homogeneity of variances, by using the statistical package SPSS 19.0 (SPSS Inc., Chicago, IL, USA). Differences between means were considered significant at *p* < 0.05.

## References

[1] Giacomini E., Rupiani S., Guidotti L., Recanatini M., Roberti M. (2016). The Use of Stilbene Scaffold in Medicinal Chemistry and Multi- Target Drug Design. Curr. Med. Chem..

[2] Berman A.Y., Motechin R.A., Wiesenfeld M.Y., Holz M.K. (2017). The therapeutic potential of resveratrol: A review of clinical trials. NPJ Precis. Oncol..

[3] Ramírez-Garza S.L., Laveriano-Santos E.P., Marhuenda-Muñoz M., Storniolo C.E., Tresserra-Rimbau A., Vallverdú-Queralt A., Lamuela-Raventós R.M. (2018). Health Effects of Resveratrol: Results from Human Intervention Trials. Nutrients.

[4] Wenzel E., Somoza V. (2005). Metabolism and bioavailability oftrans-resveratrol. Mol. Nutr. Food Res..

[5] Walle T. (2011). Bioavailability of resveratrol. Ann. N. Y. Acad. Sci..

[6] Intagliata S., Modica M.N., Santagati L.M., Montenegro L. (2019). Strategies to Improve Resveratrol Systemic and Topical Bioavailability: An Update. Antioxidants.

[7] Hoshino J., Park E.-J., Kondratyuk T.P., Marler L., Pezzuto J.M., Van Breemen R.B., Mo S., Li Y., Cushman M. (2010). Selective Synthesis and Biological Evaluation of Sulfate-Conjugated Resveratrol Metabolites. J. Med. Chem..

[8] Kim C.-W., Hwang K.-A., Choi K.-C. (2016). Anti-metastatic potential of resveratrol and its metabolites by the inhibition of epithelial-mesenchymal transition, migration, and invasion of malignant cancer cells. Phytomedicine.

[9] Giménez-Bastida J.A., Ávila-Gálvez M.Á., Espin J.C., González-Sarrías A. (2019). Conjugated Physiological Resveratrol Metabolites Induce Senescence in Breast Cancer Cells: Role of p53/p21 and p16/Rb Pathways, and ABC Transporters. Mol. Nutr. Food Res..

[10] Peñalver P., Belmonte-Reche E., Adán N., Caro M., Mateos-Martín M.L., Delgado M., Gonzalez-Rey E., Morales J.C. (2018). Alkylated resveratrol prodrugs and metabolites as potential therapeutics for neurodegenerative diseases. Eur. J. Med. Chem..

[11] Pannu N., Bhatnagar A. (2019). Resveratrol: From enhanced biosynthesis and bioavailability to multitargeting chronic diseases. Biomed. Pharmacother..

[12] Calamini B., Ratia K., Malkowski M.G., Cuendet M., Pezzuto J.M., Santarsiero B.D., Mesecar A.D. (2010). Pleiotropic mechanisms facilitated by resveratrol and its metabolites. Biochem. J..

[13] Lasa A., Churruca I., Eseberri I., Andrés-Lacueva C., Portillo M.P. (2012). Delipidating effect of resveratrol metabolites in 3T3-L1 adipocytes. Mol. Nutr. Food Res..

[14] Eseberri I., Lasa A., Churruca I., Portillo M.P. (2013). Resveratrol Metabolites Modify Adipokine Expression and Secretion in 3T3-L1 Pre-Adipocytes and Mature Adipocytes. PLoS ONE.

[15] Eseberri I., Lasa A., Miranda J., Gracia A., Portillo M.P. (2017). Potential miRNA involvement in the anti-adipogenic effect of resveratrol and its metabolites. PLoS ONE.

[16] Kapetanovic I.M., Muzzio M., Huang Z., Thompson T.N., McCormick D.L. (2011). Pharmacokinetics, oral bioavailability, and metabolic profile of resveratrol and its dimethylether analog, pterostilbene, in rats. Cancer Chemother. Pharmacol..

[17] Dellinger R.W., Garcia A.M., Meyskens F.L. (2014). Differences in the glucuronidation of resveratrol and pterostilbene: Altered enzyme specificity and potential gender differences. Drug Metab. Pharmacokinet..

[18] Peñalver P., Zodio S., Lucas R., De-Paz M.V., Morales J.C., Rodriguez R.L. (2020). Neuroprotective and Anti-inflammatory Effects of Pterostilbene Metabolites in Human Neuroblastoma SH-SY5Y and RAW 264.7 Macrophage Cells. J. Agric. Food Chem..

[19] Wang P., Sang S. (2018). Metabolism and pharmacokinetics of resveratrol and pterostilbene. BioFactors.

[20] Miksits M., Maier-Salamon A., Aust S., Thalhammer T., Reznicek G., Kunert O., Haslinger E., Szekeres T., Jaeger W. (2005). Sulfation of resveratrol in human liver: Evidence of a major role for the sulfotransferases SULT1A1 and SULT1E1. Xenobiotica.

[21] Hijona E., Bujanda L., Portillo M.D.P., Aguirre L., Palacios-Ortega S., Fernández-Quintela A. (2019). Pterostilbene Reduces Liver Steatosis and Modifies Hepatic Fatty Acid Profile in Obese Rats. Nutrients.

[22] Shao X., Chen X., Badmaev V., Ho C.-T., Sang S. (2010). Structural identification of mouse urinary metabolites of pterostilbene using liquid chromatography/tandem mass spectrometry. Rapid Commun. Mass Spectrom..

[23] Azzolini M., La Spina M., Mattarei A., Paradisi C., Zoratti M., Biasutto L. (2014). Pharmacokinetics and tissue distribution of pterostilbene in the rat. Mol. Nutr. Food Res..

[24] Remsberg C.M., Yáñez J.A., Ohgami Y., Vega-Villa K.R., Rimando A.M., Davies N.M. (2008). Pharmacometrics of pterostilbene: Preclinical pharmacokinetics and metabolism, anticancer, antiinflammatory, antioxidant and analgesic activity. Phytotherapy Res..

[25] Jay A.G., Hamilton J.A. (2018). The enigmatic membrane fatty acid transporter CD36: New insights into fatty acid binding and their effects on uptake of oxidized LDL. Prostaglandins Leukot. Essent. Fat. Acids.

[26] Bradford M.M. (1976). A rapid and sensitive method for the quantitation of microgram quantities of protein utilizing the principle of protein-dye binding. Anal. Biochem..

[27] Gillies R., Didier N., Denton M. (1986). Determination of cell number in monolayer cultures. Anal. Biochem..

[28] Laemmli U.K. (1970). Cleavage of Structural Proteins during the Assembly of the Head of Bacteriophage T4. Nat. Cell Biol..

[29] Lynen F. (1969). [3] Yeast fatty acid synthase. Methods Enzymol..

